# Antimicrobial Activity of Films and Coatings Containing Lactoperoxidase System: A Review

**DOI:** 10.3389/fnut.2022.828065

**Published:** 2022-03-04

**Authors:** Mojtaba Yousefi, Amene Nematollahi, Mahdi Shadnoush, Amir M. Mortazavian, Nasim Khorshidian

**Affiliations:** ^1^Food Safety Research Center (Salt), Semnan University of Medical Sciences, Semnan, Iran; ^2^Department of Food Safety and Hygiene, School of Health, Fasa University of Medical Sciences, Fasa, Iran; ^3^Department of Clinical Nutrition, Faculty of Nutrition Sciences and Food Technology, National Nutrition and Food Technology Research Institute, Shahid Beheshti University of Medical Sciences, Tehran, Iran; ^4^Department of Food Science and Technology, Faculty of Nutrition Sciences and Food Technology, National Nutrition and Food Technology Research Institute, Shahid Beheshti University of Medical Sciences, Tehran, Iran; ^5^Department of Food Technology Research, Faculty of Nutrition Sciences and Food Technology, National Nutrition and Food Technology Research Institute, Shahid Beheshti University of Medical Sciences, Tehran, Iran

**Keywords:** lactoperoxidase, film, coating, antimicrobial, enzyme, preservation

## Abstract

The production of safe and healthy foodstuffs is considered as one of the most important challenges in the food industry, and achieving this important goal is impossible without using various processes and preservatives. However, recently, there has been a growing concern about the use of chemical preservatives and attention has been focused on minimal process and/or free of chemical preservatives in food products. Therefore, researchers and food manufacturers have been induced to utilize natural-based preservatives such as antimicrobial enzymes in their production. Lactoperoxidase, as an example of antimicrobial enzymes, is the second most abundant natural enzyme in the milk and due to its wide range of antibacterial activities, it could be potentially applied as a natural preservative in various food products. On the other hand, due to the diffusion of lactoperoxidase into the whole food matrix and its interaction and/or neutralization with food components, the direct use of lactoperoxidase in food can sometimes be restricted. In this regard, lactoperoxidase can be used as a part of packaging material, especially edible and coating, to keep its antimicrobial properties to extend food shelf-life and food safety maintenance. Therefore, this study aims to review various antimicrobial enzymes and introduce lactoperoxidase as a natural antimicrobial enzyme, its antimicrobial properties, and its functionality in combination with an edible film to extend the shelf-life of food products.

## Introduction

Food safety (the opposite of food risk) is defined as the absence of chemical, microbial, or physical hazards in consuming foods (1). Microbial spoilage and foodborne pathogens are of great concern for the industry, authorization agencies, the health sector, and the consumer. They can lead to food deterioration and foodborne diseases, and even death in the population, in addition to restricting food shelf-life, worldwide losing production, and a public trust in the food industry (1–5). This deterioration is caused mainly by *Listeria monocytogenes, Staphylococcus aureus, Bacillus cereus*, and *Escherichia coli* through the consumption of most foods, including dairy products, meat products (especially poultry), fresh fruit and vegetable produces, and sea foods as well (6). They are frequently present in food products mainly due to post-processing recontamination during cold storage and handling (7–9). To this end, there is an imperative need to apply approaches to control the noted microbial growth in the foods (10–13).

Traditionally, several methods, including cooling, freezing, pasteurization, canning, modified atmosphere packaging (MAP), and the utilization of chemical preservatives in different fresh food products, are ascertained, which mean that the shelf-life of foods is increased and the growth of microorganisms causing human health risks is prevented (9). However, several disadvantages like high cost, a possible injury to food quality, and a potential negative impact of synthetic preservatives on human health have been observed in the last few decades (14).

In recent years, consumers have a tendency to use natural bio-preservatives (antimicrobial compounds) due to new concerns about the safety of synthetic preservatives (15–19). Today, researchers have focused on using antimicrobial agents as an efficient approach to decrease or postpone the growth and proliferation of spoilage and pathogenic microorganisms in different foodstuffs, in addition to preserve nutritional quality and increase the shelf-life of food products (2, 20–22). In this regard, organic acids, natural herbal extracts especially essential oils, bacteriocins like lacticin, nisin, and pediocin, and antimicrobial enzymes such as lysozyme, chitinase, glucose oxidase, and lactoperoxidase system (LPOS) have been considered as suitable natural antimicrobial compounds for the conservation of various foodstuffs (9, 23–25). Nevertheless, a direct use of these compounds (by dipping, dusting, and spraying) has some restrictions because the active agents could be neutralized, vaporized, or diffused rapidly into the whole food matrix and possibly react with food components (3, 26). Consequently, their antimicrobial activity against spoilage and pathogenic microorganisms may decrease during storage time and subsequently restrict their application in the food industry (3, 14).

Recently, the development of packaging (plastic or edible films and coatings) for the entrapment of antimicrobial compounds (as a kind of active packaging) could be a promising alternative approach to extend food shelf-life and food safety maintenance without affecting food's sensory characteristics (10). In addition to the effectiveness of active packaging as a good barrier to gas (oxygen and carbon dioxide), moisture, and solute migration, films and coatings could successfully decrease the microbial growth in food products by reducing the diffusion amount of antimicrobial compounds from coating and film materials into the food medium through a controlled release of antimicrobial agents (14). Therefore, lower contents of antimicrobial agents would be required in edible coatings and films to provide food conservation compared to a direct application (14). However, because of the consumers' health concerns related to the use of plastic packaging materials, there is an urgent need to use edible or biodegradable films or coating (23). Thus, antimicrobial bio-packaging received a growing interest in the food packaging industry due to their higher acceptance by the increasing “natural,” “healthier,” and “higher-quality” food concepts along with the need for an extended shelf-life (27, 28).

Edible films and coatings can be produced from diverse natural constituents, such as polysaccharides (alginate, chitosan, carrageenan, pectin, and cellulose derivatives), proteins (whey protein isolate, zein, and gelatin), and lipids (waxes) alone or in combination, taken from renewable agricultural sources or food processing wastes (14, 29–31). Several types of research have proven the ability of films and coatings containing different natural antimicrobial compounds to maintain the quality and to intensify the safety of a wide range of food products, especially highly perishable foodstuffs like fruits, vegetables, and meat products (7, 8, 24, 26, 32–34).

The LPOS is a natural antimicrobial enzyme system in milk, tears, and saliva (35–40). This system contains three principal elements: (1) lactoperoxidase enzyme (LPO), (2) hydrogen peroxide (H_2_O_2_), and (3) thiocyanate (SCN). LPO catalyzes the oxidation of SCN^−^ ion, creating oxidizing products like hypothiocyanite (OSCN^−^) and hypothiocyanous acid (HOSCN), with the aid of H_2_O_2_ (6, 7). An antimicrobial activity of the LPOS is supposed to arise from the oxidation of sulfhydryl (SH) groups present in microbial proteins and enzymes *via* these intermediate oxidizing products leading to a change in cellular functions, such as membrane integrity, passage systems, and metabolic enzymes and subsequent death of the cells (6, 15, 27, 41). It is well-known that this system has bacteriostatic and bactericidal activities against Gram-positive and Gram-negative foodborne organisms, respectively (16). Furthermore, it shows antifungal and antiviral performances (23).

The incorporation of the LPOS, generally recognized as a safe (GRAS) compound, into different kinds of edible coatings and films to control the food spoilage originated from pathogenic microorganisms has been investigated in recent years (1, 5, 15, 28, 42). However, to the best of our knowledge, there is no review regarding the antimicrobial activity of films and coatings containing LPOS in different food products. Therefore, the principal objectives of this review are to discuss the antimicrobial enzymes, physicochemical, and antimicrobial properties of the LPOS. Moreover, this study reviews the potential use of films and coatings as polymeric matrices for LPOS incorporation into food products to preserve food quality.

## Antimicrobial Enzyme

There are two main purposes associated with food preservation including decreasing the prevalence of foodborne diseases resulting from food pathogens and stopping or postponing microbial spoilage. The latter causes the food unpleasant but not essentially unsafe for consumers (43–46). Concerns about chemical preservatives increased the tendency to natural antimicrobial compounds extracted from animals, plants, and microorganisms (47).

Enzymes are highly specific and efficient in minor concentrations and are working in mild temperature and pH conditions. Furthermore, they react quickly, making them a cost-effective approach in food processing. Enzymes have been experientially consumed since very old ages. They are mainly extracted from natural sources (i.e., plants, animals, and microbial sources) and may be simply inactivated following the occurrence of an appropriate transformation. So, they are considered to be natural and safe food components that are more desirable than chemical compounds, as food-processing agents, by producers and consumers. Regarding the enormous number of enzymes successfully employed in the food industry (as biological catalysts for speeding up chemical reactions like hydrolysis, synthesis, and biocatalysis), it is reasonable to respect the potential of enzymes in the food preservation sector (43). Antimicrobial enzymes are widespread in the environment and play an important role in protecting livelihood invasion by several microorganisms, particularly bacteria and fungi (48). Recently, they are achieving distinct attention as novel and unique agents in food preservation due to antimicrobial and antibiofilm activity (49). They can extend the shelf-life of food products by inhibiting the growth of spoilage microorganisms through several mechanisms, involving decreased necessary nutrient for microbial growth, the production of bacteriostatic or bactericidal substances, and damaging cell wall and/or the inactivation of a vital enzyme in microorganisms (50). In this respect, there are several natural antimicrobial enzyme systems in animal products, such as milk and eggs like LPOS, and lysozyme, which act as a defense system against different kinds of pathogenic microorganisms, by damaging an essential compound or creating a compound that is toxic for them (51–53).

**Figure 1:**
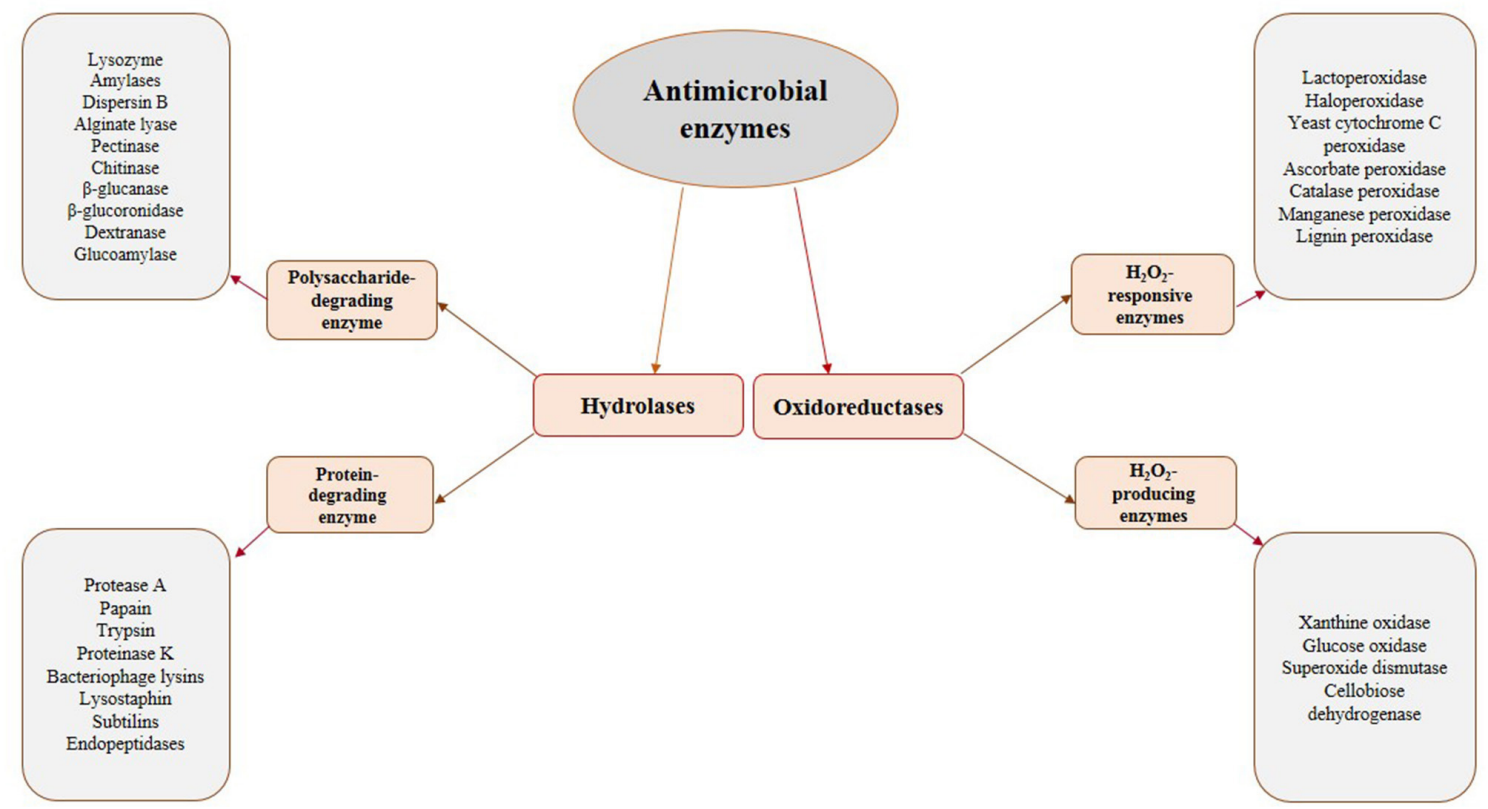
The most promising antimicrobial enzymes.

The most promising antimicrobial enzymes are shown in Figure 1. As depicted in this figure, there are two categories of antimicrobial enzymes, i.e., the hydrolases and the oxidoreductases. It is worthy to mention that the microorganisms' cell walls are the substrates for the hydrolases and their destruction could inactivate the cells. Given the significant differences in the cell wall morphology of bacteria and fungi, the potential substrates for these kinds of enzymes differ for these two classes of microorganisms. Therefore, they are classified as bacteriolytic enzymes and antifungal enzymes. The cell wall of bacteria is mainly composed of peptidoglycan (a carbohydrate backbone containing N-acetyl-D-glucosamine and N-acetylmuramic acid linked by peptide bonds), which is effective in the firmness of the bacterial cell. Cell wall deprivation by bacteriolytic enzymes may lead to cell lysis due to the osmotic pressure inside the cell. It is worthy to note that Gram-negative bacteria are less sensitive to these enzymes than Gram-positive bacteria due to the performance of the outer membrane present in the Gram-negative bacteria as an obstacle to infusion. The fungal cell wall is mainly composed of chitin, a polysaccharide created by N-acetyl-D-glucosamine and glucosidic acid linkages. Therefore, antifungal enzymes, which are effective on this structure, are possibly applicable against several foodborne fungi, similar to bacteriolytic enzymes (43). Lytic enzymes are usually classified into two groups, namely, proteases and polysaccharide hydrolases. N-acetylhexosaminidases (like Lysozyme), N-acetylmuramyl-L-alanine amidases, and endopeptidases are the main examples of this group varying in construction, target substrate, reaction process, and several physicochemical characteristics (54). Generally, the combination of these two types of hydrolyzing enzymes achieves a better antimicrobial function (54).

Unlike hydrolases, oxidoreductases, including glucose oxidase and LPOS, show their influence by the creation of cytotoxic components, which can damage key elements (especially proteins) in the microorganisms cells (43). This group is classified into two distinct categories: (1) H_2_O_2_-producing enzymes (oxidases) and (2) H_2_O_2_-responsive enzymes (peroxidases). Oxidases, like glucose oxidase, generate H_2_O_2_ in the absence of catalase or peroxidases, which could be lethal due to the toxicity of generated H_2_O_2_ and the consumption of oxygen necessary for aerobic microorganisms (50). H_2_O_2_ kills microbial cells *via* the peroxidation and degradation of the cell membrane and the prevention of protein and DNA synthesis. It is reported that low levels of H_2_O_2_ (up to 3%) are efficient as antimicrobial compounds against different kinds of food spoilage microorganisms and food borne pathogens (54, 55). H_2_O_2_-responsive enzymes mainly include haloperoxidases, myeloperoxidase (MPO), and LPO, present in several plants, animals, and humans. They use H_2_O_2_ as a substrate to oxidize halides (such as I_2_, Cl_2_, and Br_2_) or pseudohalide (like thiocyanate) to more strong antimicrobial agents (like OSCN^−^ in the case of LPOS) (54, 56). It is worthwhile to mention that the combination of the LPOS and glucose oxidase could also be a useful approach. In such conditions, glucose oxidase consumes glucose to produce H_2_O_2_ needed by the LPOS to oxidize thiocyanate (54). Table 1 shows the sources, mechanisms of action, and the microorganisms affected by several antimicrobial enzymes having potential food applications.

**Table 1:** The mechanism of an antimicrobial activity of the most important enzymes.

**Antimicrobial enzyme**	**Sources**	**Mechanism of action**	**Affected microorganisms**	**References**
Lysozyme	Hen egg white, tears, saliva, blood serum, milk, certain bacteria and bacteriophages	Hydrolyzing 1,4-beta-linkages in the peptidoglycan of the bacterial cell walls, alkaline nature	Gram-positive bacteria, Gram-negative bacteria in combination with other treatments	(58)
Chitinase	Plants and microorganisms such as streptomycetes, bacilli, and most fungi	Hydrolyzing glycosidic bonds in fungal cell-wall (chitin)	Pathogenic Fungi, some bacteria	(43)
B-glucanase	Plants and microorganisms	Hydrolyzing B (1–3) glycosidic linkages in fungal cell-wall (chitin)	Fungi	(43, 54)
Mannanase and o-glucanases	Plants and microorganisms	Hydrolyzing fungal cell-wall (chitin)	Fungi	(43)
Glucose oxidase	Molds such as *Aspergillus niger* and *Penicillium* spp.	Lowering of pH, depletion the glucose needed for microorganisms growth via production of D-gluconic acid, formation of H_2_O_2_	Food spoiling microorganisms and food borne pathogens	(55)
Lactoperoxidase	Milk, tears and saliva	Catalyzing the oxidization of thiocyanate to hypothiocyanate or higher oxyacids which react with protein sulfhydryls, by using H_2_O_2_	Gram-negative and Gram-positive bacteria, fungi, viruses	(15, 41)
Haloperoxidase	*Caldariomyces fitmago, Curvularia verruculosa*	Oxidizing halides, in the presence of H_2_O_2_, Producing HOC	Bacteria	(43, 47)
Xanthine oxidoreductase	Milk	Synthesis of free radicals, H_2_O_2_, and antioxidant compounds	Bacteria	(110)
Cellobiose dehydrogenase	Wood degrading fungi (*Myriococcum thermophilum*)	Oxidizing cellobiose and other oligosaccharides to produce H_2_O_2_, lowering the pH by production of cellobionic acid	Bacteria such *as S. aeureus, B. subtilis, Pseudomonas putida, E. coli* and *Cellulomonasmicrobium cellulans*	(48)
Subtilisins	*Bacillus* sp.	Cleaving proteins in which serine serves as the nucleophilic amino acid, hydrolyzing adhesins (bacterial proteins essential for attachment onto solid supports and other bacteria)	Wide spectrum of bacteria	(54)
Lysostaphin	*Staphylococcus simulans*	Cleaving pentaglycine cross-bridge found in the S. aureus cell wall peptidoglycan	*S. aureus* and *S. epidermidis*	(54, 111)
Papain	Plants	Hydrolyzing the peptidoglycan of the bacterial cell walls by esterase and amidase activities	Gram-negative and Gram-positive bacteria, fungi	(112)
Dispersin B	*Actinobacillus actinomycetemcomitans*	Hydrolyzing β-1,6-N-acetyl-d-glucosamine	Gram-positive and Gram-negative bacteria	(54)
Alginate lyase	Algae, invertebrates and microorganisms	Cleaving β-glycosidic bonds of bacteria alginate polymer, removing the negative charge on the carboxylate anion, abstraction of the proton on C5, and β-elimination of the 4-O-glycosidic bond (lyase)	*P. aeruginosa*	(54)

Despite several advantages of antimicrobial enzymes, the main disadvantage is sensitivity at different conditions like temperatures, salt, and pH, which could result in enzyme denaturation and inhibiting their activity (52). Therefore, the preservation of enzymes activity is essential for achieving the advantages of antimicrobial enzymes (43). Recently, the incorporation of antimicrobial enzymes, especially the LPOS and lysozymes, in active packagings, like cellulose, gelatin, and chitosan edible coatings and films, is a promising method to retain the catalytic activity of enzymes to inhibit food spoilage microorganisms (52, 54, 57, 58). It is worthy of mentioning that a few antimicrobial enzymes, including lysozyme and the LPOS, are listed as GRAS and approved for incorporation into food products (14).

## Lactoperoxidase System

The LPOS is a natural antibacterial system in milk and human saliva. This system is made of three components, including lactoperoxidase (LPO), H_2_O_2_, and thiocyanate (SCN^−^) and it is only active when all of these three compounds exist together (49, 59, 60). H_2_O_2_ and SCN^−^ originate from hepatic and cellular metabolism (61, 62). In this system, the oxidation of thiocyanate by H_2_O_2_ is catalyzed by LPO. It results in the appearance of intermediate products such as hypothiocyanate ion, sulfurdicyanide (HO SCN), and cyanosulfurous acid (HO SCN) with an antimicrobial activity against bacteria, fungi, and viruses (63, 64).

## Lactoperoxidase

Oxidative enzymes such as peroxidases exert an antimicrobial and antibiofilm activity by utilizing H_2_O_2_ to oxidize isocyanate and halides to stronger antimicrobial agents against invasive microorganisms (54, 65–67). Mammalian peroxidases differ from plant peroxidases in size, amino acid composition, the nature of the prosthetic group, and its binding to the protein. Mammalian peroxidases consist of ~576–738 amino acids with a covalently bound heme moiety, whereas there are nearly 300 amino acids with a non-covalently bound heme moiety in plant peroxidases (59). Lactoperoxidase, MPO, eosinophil peroxidase (EPO), and thyroid peroxidase (TPO) can be named as the family of mammalian heme-containing peroxidase enzymes (68).

Lactoperoxidase enzyme (EC 1.11.1.7), a heme-containing chain glycoprotein and as a member of the mammalian peroxidase family, is a natural enzyme secreted by mammalian glands and found in tears, saliva, and milk (41, 69). It is synthesized in the infants' gastrointestinal tract. It is also the enzyme that is synthesized by the mammary gland in the highest amount to protect the glands against pathogens (68, 70).

Peroxidases secreted in the lacrimal, salivary, and mammary glands are chemically and immunologically similar (71). LPO was named so because it was first isolated from milk in a crystalline form. Due to the similarity of mammalian peroxidase, other animal and human peroxidases are mostly called lactoperoxidase (68, 72). LPO is the second most abundant natural enzyme present in milk and has a great role in milk preservation, especially, in places where there is no possibility or impossibility of rapid cooling or refrigeration of milk after obtaining it (68, 73). LPO exhibits a bactericidal effect on Gram-negative and Gram-positive bacteria and antifungal and antiviral properties (2). Due to its wide range of antibacterial activities, LPO was investigated for its potential role as a natural preservative in foods and milk and in medicine (49, 74, 75).

Lactoperoxidase enzyme, a glycoprotein with 8–10% carbohydrate, consists of a single peptide chain containing 612 amino acids with a molecular weight of ~78 kDa (49, 76, 77). Bovine LPO is a basic protein with a high isoelectric pH value of 9.2, and human LPO is moderately cationic with an isoelectric pH value of 7.5 (71). At least ten fractions of LPO are recognized, and there is no considerable difference in the activity of various LPO fractions (49).

Protoporphyrin IX as the heme group is present in the catalytic center of the LPO and covalently bound to the polypeptide chain through a disulfide bridge, indicating the absence of free thiol groups present in the enzyme molecule (78–80). As a part of the heme group, the iron compound constitutes 0.07% of the LPO. Molecular conformation and structural integrity of the protein are stabilized by the calcium ion that is tightly bound to LPO (71, 78). As LPO is one of the most stable enzymes against heat, it is usually utilized as an indicator of pasteurization process efficiency (81). Furthermore, it is stable to the acidity up to pH 3 and proteolytic action of gastric juice. However, LPO is inactivated by light in the presence of riboflavin and oxygen or by the overgrowth of microorganisms. Furthermore, an excess of H_2_O_2_ causes an irreversible inactivation of LPO (64, 71).

## The Thiocyanate Ion (SCN^−^)

The thiocyanate anion is ubiquitously distributed in tissues and secretions of mammals. It is present in various glands and organs such as thyroid, salivary, and mammary glands and their secretion as well as the kidney and stomach and in biological fluids, including lymph, plasma, cerebral, cervical, spinal, and synovial fluids (72, 82, 83). Various factors such as eating and smoking habits of man affect the concentration of SCN^−^ in humans. Furthermore, the content of SCN^−^ in bovine milk depends on the breed, species, diet, health of udder, season, and geographic regions (71, 84). The content of SCN^−^ present in animal metabolism is largely dependent on the food supplied, as two major dietary sources such as glucosinolates and cyanogenic glycosides resulting in a higher amount of SCN^−^ (84).

It has been reported by Fweja et al. that 1–35 mg of thiocyanate per liter is in the fresh milk, which is not always adequate to activate the LPOS (85). Therefore, as determined by the Codex Alimentarius Commission (CAC), ~10 mg of SCN^−^ per liter should be added to raw milk to exogenously activate the system (86). On the other hand, it has been reported that human saliva and human gastric juices contain 50–300 and 40–50 ppm of thiocyanate, respectively, which are much more than the values (15 ppm) necessary for the activation of the LPOS (49, 87). The excess SCN^−^ in the body has shown to exert toxic effects and interferes with iodine metabolism causing goiters (64). However, clinical experiments have demonstrated that the treated milk with thiocyanate does not reach the limits needed to affect thyroid function (84, 88). Iodine metabolism is interfered when the thiocyanate concentration is more than 20 ppm in the human plasma (89).

## Hydrogen Peroxide

Hydrogen peroxide is the third component of the LPOS, and it is not generally found in raw milk (64, 90). Polymorphonuclear leucocytes may endogenously produce this component in the process of phagocytosis. H_2_O_2_ can also be produced by numerous microorganisms such as *Lactobacilli, Streptococci*, and *Lactococci* under aerobic conditions in adequate amounts to activate the LPOS (49, 91, 92). H_2_O_2_ generator systems such as sodium percarbonate and glucose oxidase can also produce H_2_O_2_ (71). As mentioned previously, H_2_O_2_ is naturally found in raw milk at very low concentrations. Therefore, it is necessary to add H_2_O_2_ exogenously to activate LPO (93, 94). H_2_O_2_ is very toxic to mammalian cells, but the cells are protected against toxicity when H_2_O_2_ is utilized at a low level in the presence of LPO and SCN^−^ (64, 88). Based on the criteria set by the CAC, the addition of 8.5 ppm of H_2_O_2_ is necessary for an exogenous activity of the LPOS (86), while for preserving milk using only H_2_O_2_, nearly 500–800 ppm of this component are required (64). It has been indicated that the application of glucose oxidase/glucose and xanthine oxidase/hypoxanthine as H_2_O_2_-producing systems may result in a more efficient antimicrobial LPOS than the addition of H_2_O_2_ only (71, 95).

## Modes Of Action And An Antimicrobial Spectrum Of The LPOS

As mentioned before, LPO participates in the peroxidation of thiocyanate and some halides such as I_2_, Br_2_ to produce some intermediate components that kill or prevent the growth of various microorganisms. The peroxidative reactions are complex, and they depend on the content of H_2_O_2_, the presence or absence of endogenous electron donors, and the occurrence of various pathways (49, 59, 91).

In the first step of an enzymatic mechanism, an initiation reaction of the resting LPO (Fe^+3^) to its ground state using H_2_O_2_ occurs. Following this reaction, propagation reactions take place. These reactions involve converting the ground state of LPO into the so-called compound I state by a reaction with H_2_O_2_. At low concentrations of SCN^−^ (<3 μM) and halide, compound I reacts with existing one-electron donors such as proteins and peptides, to create compound II that is consistently reduced to the ground state at a low rate. Compound II may react with excess H_2_O_2_ (>5 mM) and form compound III resulting in ferrylperoxidase adduct and an irreversible inactivation of LPO. Compound I created from the reaction of peroxidase with H_2_O_2_ participates in the oxidation of SCN^−^ and halides (49, 59, 71, 91). The oxidation of SCN^−^ results in the formation of short-lived oxidation products that participate in an antimicrobial activity of the LPOS. OSCN^−^ is the main intermediate oxidation product of SCN^−^. Furthermore, based on the reaction conditions, other short-lived intermediates such as thiocyanogen [(SCN)_2_], cyanogen thiocyanate (NC-SCN), cyanosulfurous acid (HO_2_SCN), and cyanosulfuric acid (HO_3_SCN) may be formed (49, 96, 97). OSCN^−^ is in equilibrium with HOSCN and at the pH value of 5.3 that LPO shows the maximum activity, the amount of these two forms are equal (71, 98). Both forms show an antimicrobial activity; however, the acid form is more bactericidal. Uncharged HOSCN has more solubility in the non-polar media and can more efficiently pass through the cell membrane (59, 99). On the other hand, the basic form of OSCN^−^ is more stable than its acid form. The stability of OSCN^−^ is affected by various factors such as the presence or absence of LPO, metal ions (Fe, Ni, Cu, Mn, etc.), light, pH, glycerol, and ammonium sulfate (49, 59, 100).

**Figure 2:**
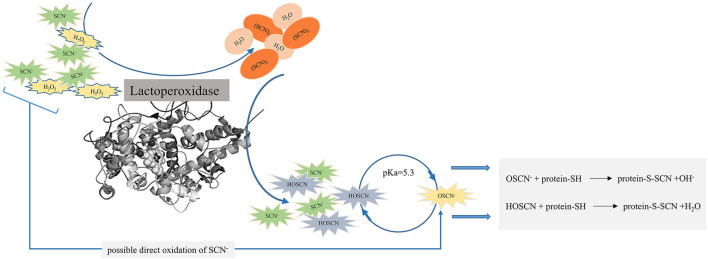
Antimicrobial activity of lactoperoxidase enzyme (LPO) by the oxidation of sulfhydryl (SH) groups of microbial enzymes and other proteins with hypothiocyanous acid (HOSCN) and hypothiocyanite (OSCN^−^).

An antimicrobial activity of the LPOS occurs through the oxidation of SH groups of microbial enzymes and other proteins with HOSCN and OSCN^−^ (Figure 2). Reducing agents containing SH groups such as cysteine, glutathione, dithiothreitol, mercapto-ethanol, and sodium hydrosulfite can prevent an antimicrobial activity of the LPOS either by scavenging thiocyanate ions or by a direct interaction with the heme group of the enzyme (49, 59, 71, 91).

The oxidation of the SH group of proteins alters proteins and enzymes such as glycolysis, respiration, and nutrient transport. Furthermore, the cell wall structure is damaged by the oxidation of the SH group, which increases its permeability and the release of important nutrients and cell components such as ions, amino acids, and peptides, inhibiting the growth of microorganisms or the occurrence of cell death (59, 70, 72, 101).

The LPOS can exert a bacteriostatic and/or bactericidal activity against various sensitive microorganisms, such as bacteria, fungi, and viruses. Based on the test medium, the type of electron donor, temperature, pH, type of microorganism, cell density and incubation time, the mechanisms of inhibitory effects of the LPOS vary from oxidative killing to the blocking of glycolytic pathways, or interfering with cytopathic effects (59, 64, 102–104).

Various groups of bacteria show a different sensitivity to the LPOS. Due to the more sensitivity of Gram-negative bacteria, the LPOS exerts a bacteriostatic effect, whereas Gram-positive bacteria are more resistant; therefore, the system has an inhibitory action only on the growth of these microorganisms (70, 102). It has been indicated that Gram-negative and catalytic organisms such as coliforms, *Salmonella, Shigella*, and *Pseudomonas* are not only prevented by LPOS but also based on the condition of media such as cell density, pH, time, and temperature of incubation, they might be killed when H_2_O_2_ is provided exogenously (49, 105). Gram-positive, catalase-negative bacteria, such as streptococci and lactobacilli are only inhibited by the LPOS (88, 106). These differences in LPOS sensitivity can be attributed to the different structures, functions, and properties of the cell wall (64, 101).

## Applications Of The Lpos In Films And Coatings

The use of edible films and coatings containing antimicrobial agents is an environmentally friendly approach for extending the shelf-life of food products. Antimicrobial edible films are preferred to direct the application of antimicrobial agents due to the controlled diffusion of these agents to food surfaces (107). Several compounds have been used as antimicrobial compounds in edible films and coatings, such as organic acids, plant extracts, bacteriocins, and enzymes (108). Table 2 represents the selected publications on the application of the LPOS in edible films and coatings.

**Table 2:** Selected publications on the application of lactoperoxidase system (LPOS) in different edible films and coatings.

**Edible film/coating**	**LPOS level**	**Microorganism**	**Main effects**	**References**
Whey protein film	0.15 g/g of film	*Salmonella enterica* and *Escherichia coli* O157:H7	Both bacteria were inhibited. Elastic modulus, tensile strength and percent elongation decreased by incorporation of 0.25 g/g LPOS, but oxygen barrier property improved by 0.15–0.25 g/g LPOS.	(26)
Whey protein film	59 mg/g of film	*Penicillium commune*	Films incorporating LPOS inhibited growth of *P*. *commune*. Elastic modulus, tensile strength, percent elongation, oxygen permeability and color values were not significantly affected by incorporating LPOS in edible film.	(8)
Whey protein coating	29 mg/g of coating	*Listeria monocytogenes*	Whey protein coating with LPOS inhibited *L. monocytogenes* in agar media and cold-smoked salmon. The tensile properties, oxygen permeability, and color of WPI films were not significantly changed by incorporation of LPOS.	(6)
Chitosan film	5% v/v in film solution	*Xanthomonas campestris* pv. *Mangifera indica, Colletotrichum gloeosporioides* (C. 64, C. 4612 and C. 62) and *Lasiodiplodia theobromae* ngr 05A	Bacterial and fungal inhibitory effect was observed. No significant change in water permeability and mechanical properties of films were detected.	(33)
Chitosan coating	5% v/v in coating solution	*Shewanella putrefaciens, Pseudomonas fluorescens*, and psychrotrophic and mesophilic bacteria	Lower bacterial growth, total volatile basic nitrogen and thiobarbituric acid values were detected in rainbow trout fillets during cold storage.	(113)
Whey protein coating	1.25, 2.5, 5 or 7.5% v/v	Aerobic mesophilic count, psychrotrophic bacteria, *Pseudomonas* spp. and specific spoilage bacteria including *Shewanella putrefaciens* and *Pseudomonas fluorescens*	High concentrations of LPOS in whey protein coating were effective in reduction of bacterial growth as well as TVBN and pH. Also, LPOS-treated samples showed higher sensory acceptability.	(2)
Chitosan coating	–	*Colletotrichum gloeosporioides, Phomopsis* sp. RP257, *Pestalotiopsis* sp. *Lasiodiplodia Theobromae* ngr 05 A	Coatings with 1 and 1.5% chitosan containing LPOS prevented fungal growth and mango ripening. LPOS had no effect on firmness, respiration, weight loss and color of mangoes.	(114)
Whey protein coating	2.5% v/v	Total viable counts, *Shewanella putrefaciens, Pseudomonas fluorescens*, Psychrotrophic bacteria	Combination of MAP and whey protein containing LPOS prevented microbial growth, reduced TBARS values and TVBN formation and maintained sensory quality for 16 days under refrigerated storage.	(115)
Alginate coating	5% v/v	*L. monocytogenes* *E. coli* O157:H7	Combination of *Zataria multiflora* Bioss essential oil and LPOS in alginate solution had the highest antibacterial effect in trout fillets.	(35)
Alginate coating	2, 4 or 6%	*Enterobacteriaceae, Pseudomonas aeruginosa*, and aerobic mesophilic	LPOS at high levels reduced the number of bacteria in chicken breast fillets and maintained the sensory properties suitably until the end of storage.	(36)
Whey protein-alginate coating	2–8% v/v	Total aerobic mesophilic bacteria, *Enterobacteriaceae*, and *Pseudomonas aeruginosa*	Coating with 8% LPOS showed the highest inhibitory activity against bacterial growth in chicken thigh meat	(5)
Chitosan coating	5%	*Phomopsis* sp. RP257 and *Pestalotiopsis* sp.	Incorporation of LPOS into chitosan solutions at 1 and 1.5% improved antifungal activity against *pestalotiopsis* sp. and *phomopsis* RP257 *in vitro* and *in vivo* conditions.	(15)
Chitosan, alginate and gelatin films	10%	Total viable count, psychrotrophic bacterial count, *Pseudomonas* spp. and *Shewanella* spp.	It was observed that chitosan films containing LPOS had the highest inhibitory activity against all bacterial growth reduced TBARS and showed the highest acceptability.	(41)

Min et al. (26) investigated the antimicrobial effect of whey protein films containing LPOS (0.15 g/g) against *Salmonella enterica* and *E. coli* O157:H7. They observed that the films inhibited *S. enterica* and *E. coli* O157:H7 (4 log CFU/cm^2^) and were inoculated either onto agar before placing the film disc or onto the top of the film disc. In a similar study, a whey protein isolate film containing 29 mg/g LPOS prevented 4.2 CFU/cm^2^ of *L. monocytogenes* inoculated on agar media. The application of LPOS-whey protein isolate coating (with 40 mg LPOS/g coating) in cold-smoked salmon decreased to near 3 and 1 log CFU/g of *L. monocytogenes* and total aerobic microorganisms, respectively. Also, whey protein coating containing LPOS inhibited *L. monocytogenes* in smoked salmon at 4 and 10°C for 35 and 14 days, respectively (6). The difference between an inhibitory activity of the LPOS in agar media and smoked salmon was attributed to a higher a_w_ of agar media that simplified the diffusion of oxidizing products from the film and a higher level of SH compounds in smoked salmon, which showed an interaction with oxidizing agents and decreased LPOS efficiency.

In a study by Lee and Min (109), defatted soybean meal was used to prepare film containing LPOS (1, 2, 3, 4, 5, and 10% w/w), which showed an inhibitory effect against Salmonella typhimurium DT 104 at the levels of 5% and 10% regardless of the inoculation level.

In another study by Min et al. (7), the LPOS at different levels (0, 3, 5, or 7% w/w) was incorporated into whey protein isolate coating and applied for roasted turkey and showed 3 and 2 log CFU/g reduction of *S. enterica* and *E. coli* O157:H7, respectively. In addition, turkey meat was inoculated with *S. enterica* (6.0 log CFU/g) or *E. coli* O157:H7 (5.6 log CFU/g), coated with whey protein containing LPOS (5 and 3% w/w) and stored at 4 and 10°C for 42 days. The results indicated an inhibitory effect against both bacteria at both temperatures during storage and the inhibition was more remarkable when the coating was formed on the surface of the turkey before inoculation than when the coating was formed on the inoculated surface.

Yener et al. (27) evaluated an antimicrobial activity of the LPOS incorporated in alginate films on *E. coli* (NRRL B-3008), *Listeria innocua* (NRRL B-33314), and *Pseudomonas fluorescens* (NRRLB-253) in the presence of different levels of H_2_O_2_ (0.2, 0.4, or 0.8 mM) and KSCN (1, 2 or 4 mM). It was indicated that the level of H_2_O_2_ and KSCN affected an antimicrobial activity of the LPOS. All bacteria were inhibited by using the LPOS along with 0.4 or 0.8 mM H_2_O_2_ and 4 mM KSCN during a 6-h incubation. Also, by applying 0.8 mM H_2_O_2_ and 4 mM KSCN, the LPOS showed an inhibitory effect against *L. innocua* and *P. fluorescens* for a 24-h incubation period. The highest and lowest resistant bacteria to the LPOS were *E. coli* and *P. fluorescens*, respectively.

The antimicrobial effects of chitosan films containing LPOS (5% v/v) on *Xanthomonas campestris* pv. *Mangifera indica, Colletotrichum gloeosporioides* (C. 64, C. 4612, and C. 62), and *Lasiodiplodia theobromae* ngr 05A were investigated. The results showed that chitosan at the levels of 1 and 1.5% containing LPOS with or without iodine completely inhibited *X. campestris* pv. *M. indica*. It was stated that, compared to Gram-negative bacteria, Gram-positive bacteria had a lower resistance to LPOS without iodine. In the case of molds, 1 and 1.5% chitosan incorporated by LPOS with or without iodine inhibited *C. gloeosporioides* C64 and *L. theobromae*, whereas *C. gloeosporioides* C4612 was susceptible to iodine and C62 was resistant (33).

Lactoperoxidase system (5% v/v) was added to chitosan coating, which was applied for the shelf-life extension of the rainbow trout. The results indicated an inhibitory activity against *Shewanella putrefaciens, P. fluorescens*, and psychrotrophic and mesophilic bacteria during a 16-day storage. Regarding the production of total volatile basic nitrogen (TVBN) and thiobarbituric acid values, lower levels were detected in LPOS-coated chitosan (113). In a similar study, Shokri et al. (2) investigated the effect of different LPOS concentrations (1.25, 2.5, 5, or 7.5%) incorporated into whey protein coating as an antimicrobial strategy for preserving the rainbow trout during a 16-day storage at 4°C. Samples treated with 7.5% LPOS showed a lower mesophilic aerobic count, psychrotrophic bacteria, *Pseudomonas* spp., and specific spoilage bacteria, including *S. putrefaciens* and *P. fluorescens* in fish filets. Total volatile basic nitrogen values were below the upper limit of acceptability for the treated samples with 2.5%, 5%, and 7.5% LPOS. pH values of LPOS-treated samples, except at 1.25% level, were lower than control samples. Regarding sensory properties, high LPOS concentrations had greater acceptability. An inhibitory effect of the LPOS against bacteria was ascribed to the presence of OSCN^−^ and HOSCN that oxidized the SH groups in the structure of enzymes and other proteins.

Cissé et al. (114) studied the effect of chitosan coating (with 0.5, 1, or 1.5% chitosan) with or without LPOS on the postharvest quality of mango. It was reported that coatings with 1 and 1.5% chitosan containing LPOS prevented fungal growth and mango ripening. However, the firmness, respiration, weight loss, and color of mangoes were not affected by the LPOS but were influenced by the chitosan level.

In a study by Rostami et al. (115), the effect of whey protein coating containing LPOS in combination with MAP on the microbiological, chemical, and sensory properties of Pike-Perch filets was determined during a 16-day of storage. It was demonstrated that whey protein coating + LPOS + MAP had the lowest bacterial count and *S. putrefaciens* and *P. fluorescens* and the total viable count as well as psychrotrophic bacteria remained below 7 log CFU/g at the end of storage. The highest and the lowest TVBN values were detected in whey protein coating and coating + LPOS + MAP, respectively. The TVBN level in samples coated with whey protein, whey protein + LPOS, and whey protein + MAP was higher than the acceptable limit of 30–35 mg N/100 g. It was noted that the use of MAP in combination with the LPOS reduced fat oxidation and consequently thiobarbituric acid reactive substances due to the decreased oxygen level. Regarding pH, samples coated with whey protein + LPOS and whey protein + LPOS showed a lower pH increase due to lower bacterial growth. Also, a sensory analysis was indicative of higher acceptability of samples coated with whey protein + LPOS + MAP during a 16-day storage. It was highlighted that in MAP packaging, replacing the oxygen with nitrogen decreased the growth of aerobic bacteria during the logarithmic phase, and the lag phase was prolonged by carbon dioxide. Similarly, Shokri and Ehsani (4) assessed the effect of whey protein coating containing 2.5% LPOS and 1.5 or 3% α-tocopherol on the quality characteristics of Pike-Perch filets during a 16-day storage at refrigeration temperature. The results demonstrated that whey protein-LPOS-coated samples had a lower bacterial count, TVBN and pH, and a higher overall acceptability. Regarding the TBA value, treatments containing only α-tocopherol had lower values than samples containing both α-tocopherol and LPOS. It was concluded that a combination of α-tocopherol and the LPOS could exhibit an antibacterial and antioxidant activity for the shelf-life extension of Pike-Perch filets during cold storage. It was also noted that α-tocopherol diminished an antimicrobial activity of the LPOS through the inhibition of SH group oxidation and the formation of disulfide bonds.

Sharifi et al. (35) studied an antimicrobial effect of alginate coating containing *Zataria multiflora* Bioss essential oil (0.5 and 1% w/v) and the LPOS (5% v/v) individually and in combination against *L. monocytogenes* and *E. coli* O157:H7 in trout filets during a 16-day storage. The lowest bacterial count was observed in samples coated with alginate solution containing 0.5% and 1% essential oil, and the LPOS was indicative of a synergistic effect of these two antimicrobial agents.

Yousefi et al. (36) investigated the effect of alginate coating with the different levels of LPOS (2, 4, or 6%) on the quality and sensory properties of chicken breast filets during a 16-day cold storage. The results showed that the pH of chicken breast filets coated with alginate-LPOS coating was lower than control samples. However, increasing the LPOS level led to an increase in pH until day 12 due to the production of H_2_O and the reduction of H^+^ concentration. Alginate-LPOS coating decreased TVBN formation in chicken filets. Chicken breast filets coated with alginate-LPOS at levels of 4% and 6% had lower *Enterobacteriaceae, Pseudomonas aeruginosa*, and aerobic mesophilic bacteria. Also, coated samples with 6% LPOS had the highest overall acceptability. The antimicrobial activity of coating containing LPOS was attributed to the interaction of OSCN^−^ and HOSCN with SH groups of proteins in the cell membrane and the release of cell components. Also, it has been reported that an uptake of glucose, purines, and pyrimidines and the production of proteins, DNA, and RNA were prevented.

In a study by Molayi et al. (5), whey protein-alginate coatings with lactoperoxidase (2, 4, 6, or 8% v/v) were applied for the control of total aerobic mesophilic bacteria, *Enterobacteriaceae*, and *P. aeruginosa* in chicken thigh meat. It was observed that increasing the level of LPOS enhanced an antibacterial activity and in this regard, coating with 8% v/v LPOS showed the highest activity. It was mentioned that the LPOS in the coating required 2 days for a complete activation against *Enterobacteriaceae*. Also, the highest inhibitory effect of the LPOS against aerobic mesophilic bacteria occurred after 4 days.

Lactoperoxidase was added at different levels (1.25, 2.5, 5, and 7.5%) to whey protein coating and its effect on the microbial, chemical, and sensory properties of shrimp was investigated and stored at the refrigerated temperature for 16 days. It was revealed that the LPOS at the level of 7.5% significantly decreased mesophilic aerobic bacteria, psychrotrophic bacteria*, Pseudomonas* spp., *P. fluorescens*, and *S. purefaciens* in shrimp samples. Coating of shrimp with whey protein and different levels of LPOS decreased TVBN formation, which remained below the unacceptable limit even after a 16-day storage. LPOS-coated samples (5 and 7.5%) showed the highest scores in different sensory parameters (9).

Saravani et al. (116) investigated the effect of whey protein coating containing LPOS (5% v/v) and *Bunium persicum* essential oil (0.5% v/v) on the microbial and chemical characteristics of Gouda cheese during a 90-day storage. It was indicated that bacteria number was constant in all essential oil and essential oil-LPOS-coated cheese samples during a 90-day storage. However, LABs and *Enterobacter* spp. were less susceptible to the antibacterial effects of LPOS. In addition, the occurrence of lipid oxidation decreased in cheese samples coated with whey protein-essential oil and whey protein-essential oil-LPOS. It was suggested to utilize LPOS with essential oil to achieve an antibacterial activity for both Gram-negative and Gram-positive bacteria. In a similar study, the LPOS (5% v/v) and *B. persicum* essential oil (0.5 or 1% w/v) were incorporated into alginate coating and its effect on *L. monocytogenes* in chicken breast filets was evaluated during a 20-day storage at 4°C. The lowest count of *L. monocytogenes* was obtained in the coated samples containing LPOS + 1% essential oil (1). It has been declared that the degree of susceptibility of bacteria was dependent on aerobiosis/anaerobiosis and growth phase and generally Gram-negative catalase-positive bacteria such as *Pseudomonas* spp. and coliforms are killed, but Gram-positive and catalase-negative bacteria, such as streptococci or lactobacilli, are inhibited by LPOS. In this regard, the LPOS is generally used along with other preservation methods.

The effect of alginate coating containing LPOS (5%), *Z. multiflora* essential oil (0.5%, 1%), or both on microbial and chemical characteristics of rainbow trout filets has been investigated during a 16-day storage. The results revealed that the combination of essential oil (1%) and LPOS (5%) was efficient in reducing mesophilic viable counts, psychrotrophic bacteria, *S. putrefaciens*, and *P. fluorescens* as well as the control of pH increase and the reduction of total volatile nitrogen. Also, the use of the LOPS individually was more effective than *Z. multiflora* essential oil alone (69).

In a study by Ehsani et al. (41), different active films including chitosan, alginate, and gelatin containing LPOS or sage essential oil were prepared, which were utilized for wrapping fish burgers and stored at 4°C for 20 days. Total viable count, psychrotrophic bacterial count, *Pseudomonas* spp. and *Shewanella* spp., TBARS, pH, and the sensory properties of the samples were determined during refrigeration. It was observed that chitosan films containing LPOS had the highest inhibitory activity against all bacterial growth. The underlying mechanisms included synergistic interactions between the components, the prevention of biochemical pathways and enzymes, and an interaction with cell wall components and pore formation in the cell wall. Also, chitosan films with LOPS reduced TBARS and showed the highest acceptability.

Mohamed et al. (15) prepared chitosan coating containing LPOS and LPOS with iodine (LPOSI) and evaluated their antifungal activity against *Phomopsis* sp. RP257 and *Pestalotiopsis* sp. isolated from mango. It was indicated that *in vitro* condition, *Pestalotiopsis* sp. was inhibited by chitosan alone at the levels of 1 and 1.5% and incorporation of the LPOS and LPOSI to 0.5% chitosan resulted in an increase of inhibition from 26 to 93%. In the case of *Phomopsis* sp. RP257, chitosan films with the LPOS and LPOSI were effective, especially at 1 and 1.5% chitosan levels. Similarly, it was stated that chitosan films completely prevented *Pestalotiopsis* sp. in mango at 1 and 1.5% while *Phomopsis* sp. RP257 inhibited 50% at the mentioned levels. Also, LPOS addition improved an antifungal activity.

## Conclusion

Recently, general trend has emerged that consumers are concerned about the safety of chemical preservatives utilized in food. Hence, extensive and integrated efforts have been carried out to discover natural-based preservatives that are able to prevent microbial growth in food and improve the safety and shelf-life of food products. This study revealed that lactoperoxidase as a natural antimicrobial enzyme in the LPOS could be potentially applied as a replacement for chemical preservatives to improve the quality and shelf-life of food products. An antimicrobial activity of LPO is supposed to arise from the oxidation of SH groups present in microbial proteins and enzymes *via* these intermediate oxidizing products leading to a change in cellular functions, such as membrane integrity, passage systems, and metabolic enzymes and subsequent death of the cells. This system has bacteriostatic and bactericidal activities against Gram-positive and Gram-negative foodborne microorganisms, respectively. Additionally, it shows antifungal and antiviral performances. However, a direct incorporation of LPOS in food matrices could lead to the reduction of its activity as a consequence of an interaction with food components and its utilization in edible films and coatings. Conducted researches in respect of films and coatings containing LPOS showed an antimicrobial activity and improved food product shelf-life and in some cases, reduced chemical spoilage in food products without a negative impact on sensory properties. Nevertheless, the types of films and coating with LPOS were limited to alginate, chitosan, gelatin, and whey protein and further researches are required to examine the antimicrobial effects of the different types of edible films and coatings incorporating LPOS. Additionally, an antimicrobial effect of the LPOS in combination with other natural antimicrobial agents and hurdle technology can be a subject of future researches.

## Author Contributions

This study was designed by NK. The manuscript was written by MY, NK, AN, MS, and AM. MY and NK critically revised the manuscript. All authors listed have approved the manuscript for publication, contributed to this article, and approved the submitted version.

## Conflict of Interest

The authors declare that the research was conducted in the absence of any commercial or financial relationships that could be construed as a potential conflict of interest.

## Publisher's Note

All claims expressed in this article are solely those of the authors and do not necessarily represent those of their affiliated organizations, or those of the publisher, the editors and the reviewers. Any product that may be evaluated in this article, or claim that may be made by its manufacturer, is not guaranteed or endorsed by the publisher.
